# Gut-Antimicrobial Peptides: Synergistic Co-Evolution with Antibiotics to Combat Multi-Antibiotic Resistance

**DOI:** 10.3390/antibiotics12121732

**Published:** 2023-12-14

**Authors:** Piyush Baindara, Santi M. Mandal

**Affiliations:** 1 Radiation Oncology, NextGen Precision Health, School of Medicine, University of Missouri, Columbia, MO 65211, USA; 2 Department of Biotechnology, Indian Institute of Technology Kharagpur, Kharagpur 721302, India; mandalsm@gmail.com

**Keywords:** gut microbiota, gut peptides, multi-antibiotic resistance, co-evolution

## Abstract

Due to huge diversity and dynamic competition, the human gut microbiome produces a diverse array of antimicrobial peptides (AMPs) that play an important role in human health. The gut microbiome has an important role in maintaining gut homeostasis by the AMPs and by interacting with other human organs via established connections such as the gut–lung, and gut–brain axis. Additionally, gut AMPs play a synergistic role with other gut microbiota and antimicrobials to maintain gut homeostasis by fighting against multi-antibiotic resistance (MAR) bacteria. Further, conventional antibiotics intake creates a synergistic evolutionary pressure for gut AMPs, where antibiotics and gut AMPs fight synergistically against MAR. Overall, gut AMPs are evolving under a complex and highly synergistic co-evolutionary pressure created by the various interactions between gut microbiota, gut AMPs, and antibiotics; however, the complete mechanism is not well understood. The current review explores the synergistic action of gut AMPs and antibiotics along with possibilities to fight against MAR bacteria.

## 1. Introduction

The rapid emergence of MAR and bacterial infections are global health concerns that urgently need to be addressed. The unavailability of new antibiotics and failure of available therapeutic strategies due to resistance development results in severe health complications and a sharp rise in deaths throughout the world [1,2]. In light of these facts, there is an urgent need for new antimicrobials and the development of new antimicrobial therapeutic strategies with effective outcomes to win the battle against MAR. AMPs are one of the promising options to fight against MAR due to their ubiquitous availability and diverse activity spectrum [3,4]. Additionally, the amenability of AMPs to bioengineering and drug repurposing may also play an important role in the development of new strategies to treat MAR [5,6,7]. Interestingly, the human gut is a complex environment where the cohabitation of pathogens with a beneficial gut microbiome and host appeases the synergistic co-evolution and action of gut AMPs and antibiotics. AMPs are also known to have multiple antimicrobial properties within a single peptide including membrane permeabilization and inhibition of both transcription and translation [8]. In the complex environment of the gut, high antimicrobial strength and complexity are observed in the tightly synchronized secretion of AMPs enriched with interdependent properties [9]. Host defense peptide-producing cells in the gut also take advantage of this synergistic action of gut AMPs in specific combinations that result in higher efficiency against pathogens at low concentrations. Similarly, the synergistic action of gut AMPs is observed with conventional antibiotics and could be used to develop new therapeutics against MAR. Interestingly, because of their known benefits, AMP-based drugs are now under consideration by the European Medicines Agency (EMA) and the Food and Drug Administration (FDA) [10,11]. Although gut AMPs have already been discussed extensively as a potential alternative to fighting against MAR, new strategies are required to control the development and evolution of rapid resistance [12,13,14]. Also, it is important to understand the synergistic and rapid evolutionary process of gut AMPs with antibiotics. Here in the present review, we discuss the use of gut microbiota-produced AMPs in conjugation with conventional antibiotics, their synergistic co-evolution, and their action in controlling MAR. 

## 2. Influence of Antibiotics on Gut Microbiota, Susceptibility to Infections, and Resistance Development

The most frequent and significant factor altering the normal gut microbiome composition and function is the use of antibiotics; however, many other factors that might impair the beneficial gut microbiota include mental and physical stress, radiation treatment, altered gut peristalsis, gastrointestinal infections, and dietary changes [15]. Antibiotics have a major impact on changing the gut microbiota, resulting in decreased bacterial diversity and increased numbers of some taxa [16]. This change in gut microbiome further results in the altered production of AMPs produced by gut microbiota and their associated functions impacting host immunity. Additionally, antibiotics’ activity spectrum, mode of action, potency, pharmacokinetics, dosage, and length of administration are also major factors that influence the gut AMPs and microbiome [17]; however, the presence of preexisting antimicrobial resistance genes in an individual’s microbiome is another concerning factor. Changes in the variety of gut bacteria can result in *Clostridium difficile* infection, which is naturally resistant to many antibiotics [18]. Other unintended consequences of antibiotic use on gut microbiota include selection for a reservoir of bacterial antibiotic resistance genes, and progression of horizontal gene transfer between bacterial strains that affects the expression, production, and regulation of gut AMPs further leading to immune dysregulation and antibiotic resistance development [16]. 

Antibiotics affect the local gut immune system by changing the composition of the gut resident microbiota and their metabolites, specifically AMPs. It has been shown that post-antibiotic treatment, the small intestine showed lower IL-17 and INF-γ production, while the colon showed decreased numbers of T_reg_ cells. This suggests that antibiotics induce altered host–microbiota interactions that cause immune imbalance [19]. Additionally, the gut microbiota stimulates mucin production, whereas antibiotics cause the weakening of the mucus barrier, making the body more vulnerable to bacterial invasion and subsequent infections [20]. Intestinal infections may be brought on by newly acquired pathogens or by the overgrowth and pathogenic potential of opportunistic microorganisms due to changes in the bacterial populations that ordinarily inhabit the gut lumen. Numerous studies on infants receiving antibiotics, particularly preterm ones, have been conducted. The normal bacterial microbiota of infants is changed by treatment with different antibiotics, such as cephalexin, gentamicin, vancomycin, and erythromycin, by increasing the percentage of potentially pathogenic *Enterobacteriaceae* and decreasing the number of bacteria like *Bifidobacteriaceae*, *Bacilli*, and *Lactobacillus* which are part of the healthy microbiota [21]. Overall, antibiotics displayed a significant role in the modulation of gut microbiota that leads to infection susceptibility at one end and resistance development as another counterpart.

## 3. Interplay of Gut Microbiota with Gut AMPs

Gut microbiota plays an essential role in the regulation of the host defense system by maintaining gut homeostasis. Bacteroidetes, Firmicutes, Actinobacteria, and Proteobacteria form the majority of the gut microbiome [22]. The diverse array of gut AMPs produced by gut microbiota plays an important role in various functional activities in the gut such as immunomodulatory activities and protection against pathogens by disrupting bacterial cell membranes and halting the RNA and DNA synthesis of metabolism [23]. Bacteriocins are the major bacterially produced gut AMPs and efficiently compete with other microbes in the gut. However, much of the gut microbiome’s diversity is still unknown; a study of some isolated microbes and metagenomic analysis suggested that there are many unrevealed classes of antibiotics and AMP-producing microbes present in the gut that are as yet unknown [24]. Gut microbiota-derived AMPs have been reported to protect against various disease-causing pathogens in the human gut (Table 1). A bacteriocin, Abp118, produced by a gut microbe *Lactobacillus salivarius* UCC118 in the gut is confirmed to protect against the foodborne pathogen *Listeria monocytogenes*. It has been confirmed that mutant *L. salivarius* UCC118, expressing the cognate Abp118 immunity protein AbpIM, failed to protect against *L. monocytogenes* infections in mice [25]. Another bacteriocin, thuricin CD, produced by *Bacillus thuringiensis* DPC 6431 has been shown to have efficient killing potential against disease-causing clinical isolates of *C. difficile* without any antagonistic effect on commensal gut microbiota [26]. Bacteriocin encoded by pheromone-responsive plasmids is common in enterococcus strains residing in the gut which are reported as gut commensals as well as for casing hospital-acquired infections [27]. Bacteriocin 21, produced by conjugative plasmid pPD1 of *Enterococcus faecalis*, is demonstrated to protect against vancomycin-resistant enterococci without affecting the other commensal microbiota in the gut. Interestingly, *E. faecalis* containing pPD1 plasmid outcompetes and replaced other *E. faecalis* lacking pPD1. This suggests that gut bacteriocin can also regulate the niche in the gut and can be used as potential therapeutic peptides able to target MAR bacteria specifically [28]. Another study showed that microcins produced by a probiotic bacterium *Escherichia coli* Nissle 1917 (EcN) can regulate inter- and intra-species competition among the *Enterobacteriaceae* and other related pathogens in the inflamed gut environment and are suggested as potential narrow-spectrum therapeutic agents against enteric pathogens [29]. 

Gut-epithelium-derived peptides are also reported to have potential antimicrobial activities against gut pathogens. In the gastrointestinal tract, enterocytes and Paneth cells are the primary cells responsible for the production of AMPs; however, macrophages, dendritic cells, neutrophils, and lymphocytes present in the lamina propria can also produce AMPs [48,49]. Defensins are the major AMPs secreted within the intestinal mucosa. The α and β defensins are abundant AMPs in the gut which are primarily secreted by Paneth and epithelial cells, respectively, in the intestine and the colon [50]. Further, it has been reported that the secretion of gut AMPs by Paneth cells is regulated and stimulated by exposure to live pathogens (both Gram-positive and Gram-negative) or bacterial products such as lipopolysaccharide, lipoteichoic acid, lipid A, and muramyl dipeptide [51]. In another study, the gut resident *Lactobacillus* population exhibited a correlation with the gene expression of α defensins, where α defensin gene expression is restored in antibiotic-treated mice by *Lactobacillus* administration. Further, it has been confirmed that α defensin gene expression by Paneth cells is regulated by commensal bacteria via the TLR-MyD88 signaling pathway that provides a deeper understanding of the involvement of gut microbiota and AMPs in gut homeostasis [52]. Overexpression of α defensin 5 is found associated with a severe reduction in the colonization of segmented filamentous bacteria that are further linked with reduced levels of Th17 cells in the lamina propia and suggests the role of α defensins in the regulation of commensal microbiota [53]. Gut epithelial-produced β defensins 2 and 3 were reported to reduce the intestinal damage caused by a gut pathogen *Salmonella typhimurium* via enhancing the probiotic activity of *Enterococcus faecium* by alteration of cytokine expression [54]. Similarly to defensins, cathelicidins are also reported to produce and act against gut pathogens by improving the gut epithelial barrier. In a recent, cathelicidin-WA has been shown to improve host defense and epithelial barrier functions by reducing enterohemorrhagic *Escherichia coli*-induced inflammation and microbiota reduction in the intestine of mice [55]. Cathelicidin-related antimicrobial peptides (CRAMP) were found to protect against an enteric pathogen, *Citrobacter rodentium*, by reducing epithelial cell damage and systemic clearance of infection [56]. In another study, cathelicidins significantly improved the gut barrier against pathogens in mouse colon mucosa where endogenous stimulation or administration of cathelicidin is able to clear the infection caused by *Escherichia coli* O157:H7 and also regulate the gut microbiota balance; this aids mucosal homeostasis [57,58]. Other major gut peptides are regenerating AMPs (RegAMP) which are soluble lectins and mainly produced by Paneth cells. A RegAMP, RegIIIγ, is demonstrated to play an important role in maintaining gut homeostasis by spatial segregation of gut microbiota and host in the intestine [59]. Another study reported that RegIIIγ can protect against *L. monocytogenes* infection via MyD88-mediated conditioning of gut epithelium [60]. Further, RegAMP is reported to have a role in pathogen clearance that is dependent on the presence of initial healthy gut microbiota and it has been suggested that gut microbiota and gut AMPs are the key factors that regulate the host response during antibiotic treatment [61]. Overall, available reports suggest a complex relationship between gut-epithelium-derived AMPs and gut microbiota; however, further studies are required to explore the regulatory switches that drive the production of gut epithelium AMPs in response to specific gut commensals or pathogens.

## 4. Synergistic Action of Gut AMPs with Conventional Antibiotics

**Figure 1:**
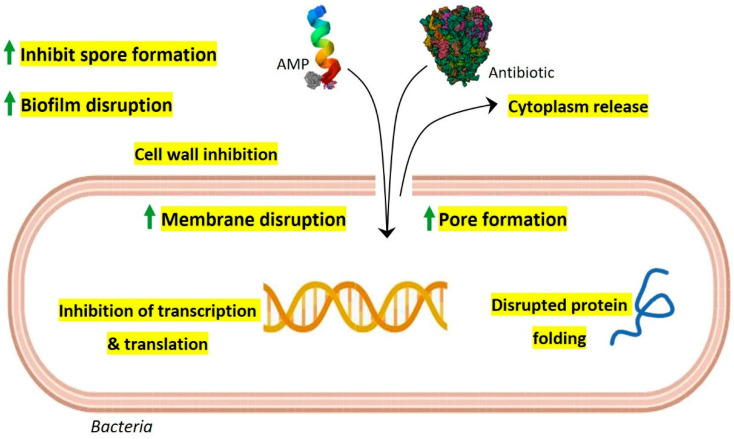
Possible model for synergistic antimicrobial activity of gut AMPs with conventional antibiotics. As per their membrane-acting properties, continuous pore formation and increased membrane permeabilization by AMPs allow more influx of antibiotics and AMPs which results in efficient bactericidal activity along with improved targeting of intracellular components such as transcription, protein synthesis machinery, and protein folding. Gut AMPs might also facilitate enhanced biofilm disruption and inhibition of spore formation when used in combination with antibiotics.

Due to the rapid emergence of multidrug resistance and the reduced efficacy of conventional antibiotics, the synergistic action of gut AMPs with antibiotics is explored and suggested as a new approach to control drug-resistant bacteria (Table 2). Interestingly, AMPs display multiple mechanisms of action at a time that include membrane pore formation, inhibition of cell wall synthesis, biofilm disruption, inhibition of spore formation, and inhibition of protein synthesis and folding, along with inhibition of DNA and RNA synthesis [12]. Especially in the complex gut environment with the possibility of numerous unknown interactions, the multiple-mode-of-action scenario of AMPs is intriguing. The synergistic action of conventional antibiotics with gut AMPs is possibly benefited by extended pore opening on the target cell membrane, increased membrane permeabilization, and subsequently increased repair time that further results in altered bacterial intracellular functions and overall bactericidal activity (Figure 1). In one of our previous studies, we have shown that laterosporulin10, a defensin-like bacteriocin produced by *Brevibacillus laterosporus* SKDU10, exhibits a synergetic effect with rifampicin against *Mycobacterium tuberculosis* H37Rv. It is confirmed that the addition of 0.25 µM laterosporulin10 results in a four-fold reduction of the rifampicin MIC values against *M. tuberculosis* [62]. Gut AMPs, nisin Z, and pediocin PA-1 including colistin were reported to have potential synergistic effects against MAR *P. fluorescens* when used in combination with antibiotics such as kanamycin, tetracycline, and chloramphenicol [63]. Nisin is also reported to have synergistic antimicrobial action with peptidoglycan-modulating antibiotics and ramoplanin, and exhibits promising activity against methicillin-resistant *S. aureus* (MRSA) and vancomycin-resistant enterococci (VRE). Furthermore, nisin demonstrates improved antibiofilm and antibacterial activity against *E. faecalis* by exhibiting synergistic effects with antibiotics such as penicillin, ciprofloxacin, and chloramphenicol [64]. A different study showed the synergistic effects of nisin with several antibiotics, including penicillin, amoxicillin, tetracycline, streptomycin, and ceftiofur against the swine pathogen *Streptococcus suis* that is known to cause severe infections in pigs [65]. Another study reported the synergic effects of subtilosin with clindamycin and metronidazole when used against *Gardnerella vaginalis*, which causes bacterial vaginosis [66]. In vitro, the activity of various human AMPs, LL-37, HBD1 to HBD3, HNP1, and HD5 have been checked against *C. difficile* in combinations of different antibiotics including tigecycline, moxifloxacin, piperacillin-tazobactam, and meropenem. Interestingly, LL-37 and HBD3 were found to have synergistic action against *C. difficile* with all the tested antibiotics [67]. Cryptdin 2, an AMP produced by Paneth cells, showed a synergistic effect against MAR *S. typhimurium* when used in combination with ampicillin [68]. HNP-1 was also confirmed to exhibit synergistic action with rifampicin and isoniazid against *M. tuberculosis* H37Rv [69]. Further, LL-37-derived membrane active analogs, FK13-a1 and FK13-a7, showed synergistic action against multidrug-resistant *P. aeruginosa* (MDRPA) and methicillin-resistant *S. aureus* (MRSA) when used in combination with chloramphenicol [70]. Next, LL-37 and colistin are reported to have synergistic action against MAR carbapenem-resistant *P. aeruginosa*, *Klebsiella pneumoniae*, and *Acinetobacter baumannii* when used in combination with the antibiotic azithromycin [71]. In a different study, short-cationic AMPs exhibited a synergistic effect with the antibiotics polymyxin B, erythromycin, and tetracycline against MDRPA [72]. A pilot study confirmed the synergistic action of colistin and the antibiotic tobramycin against *P. aeruginosa* [73]. Although multiple reports are available with improved results concerning the synergistic actions of gut AMPs with conventional antibiotics, the specific mechanisms of action need to be studied in further detail. However, in light of the evidence and analyzed results, it is possible to develop synergistic combinations of gut AMPs and antibiotics for the treatment of MAR human pathogens.

## 5. Gut AMPs, Conventional Antibiotics, and Evolution of Resistance Development

A major global public health concern is bacterial resistance to small-molecule antibiotics that are already on the market. The global spread of antibiotic-resistant bacteria has created the possibility of a post-antibiotic age in which ordinary illnesses and small wounds could develop into potentially fatal conditions. Such resistance has resulted in the creation of multidrug-resistant bacteria over the past few decades, which can both endanger healthy people and cause serious infections in immunocompromised patients. For instance, hospital-acquired infections with ampicillin-resistant *E. coli*, vancomycin-resistant *E. faecalis*, and methicillin-resistant *Staphylococcus aureus* (MRSA) have all increased in frequency [80,81]. Thus, there is an urgent need for novel antimicrobial strategies given the rising threat of MAR bacteria.

**Figure 2:**
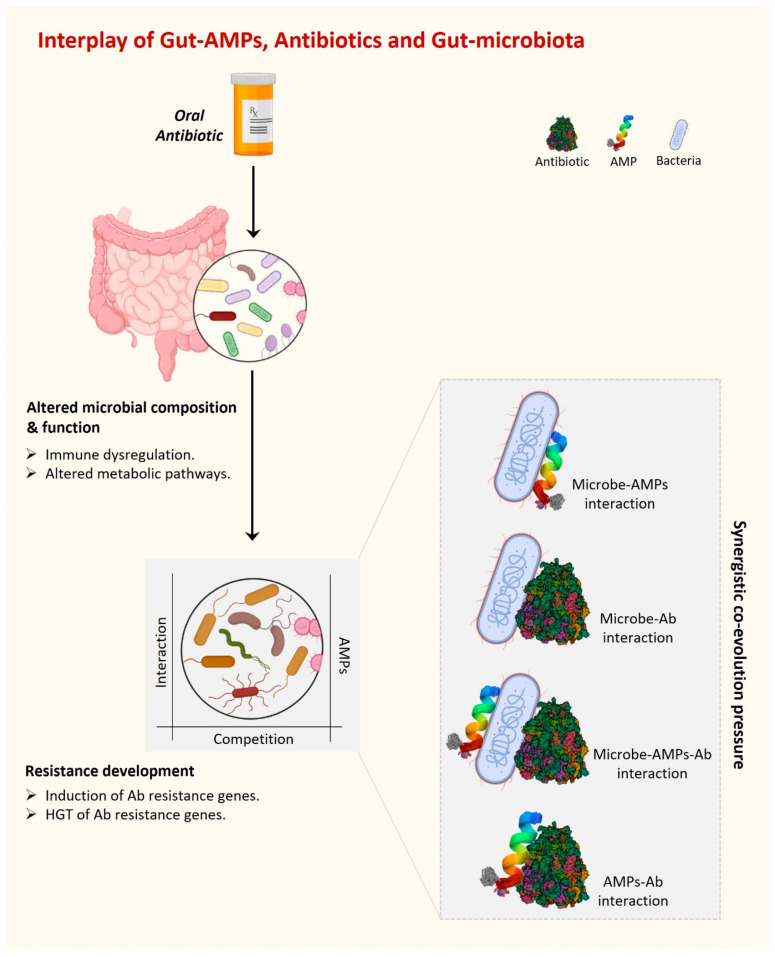
The interplay of gut AMPs, antibiotics, and gut microbiota is driven by various interactions among them. These interactions develop a synergistic co-evolutionary pressure under which gut AMPs are co-evolved to fight against MAR.

Antibiotic misuse has contributed to the emergence of MAR organisms. MAR infections are a leading source of morbidity and mortality worldwide [57]. Microbes can create and use defense and resistance mechanisms against the substances used to eradicate them in a complex environment such as the human gut, which is the home of over 100 trillion bacteria. Interestingly, not only the external antibiotics but also the antimicrobial substances produced by competitors present a challenge for the gut resident bacterial community to survive. Further, in the case of dysbiosis, an additional competition force exists between beneficial and harmful gut microbiota. AMPs are one such strategy used by bacteria (beneficial or harmful) to kill their competitors present in the surrounding complex environment. In addition to all of this, host-gut-derived AMPs are also present in the gut under the regulatory pressure of foreign AMPs, antibiotics, and the presence of their producers. Overall, there are multiple dynamic interactions present in the complex environment of the gut between various gut AMPs and antibiotics, whether internal or external. Together, these dynamic interactions and regulatory pressures create an evolutionary force under which microorganisms acquire a fair chance to evolve survival strategies and eventually develop antibiotic resistance (Figure 2). A pool of resistant genes belonging to several classes of antibiotics has been identified in a recent metagenomic study of gut resistome conducted across different continents [82]. 

Natural gut resident bacteria, bacteria with acquired resistance genes, and acquired bacteria with resistance genes that do not typically colonize the gut are all included in the gut resistance reservoir [83]. Although it is uncommon, it is conceivable for resistance genes or virulence features to be transferred between pathogenic and non-pathogenic gut resident bacteria. The interesting question of how the resident gut bacteria and gut AMPs have maintained their efficiency through evolutionary timeframes is prompted by the growing issue of antibiotic-resistant bacteria. This question may have a partial explanation in the fact that there is a huge diversity of AMPs in the gut produced by intestinal epithelial cells as well as by healthy gut microbiota, decreasing the chance of combination resistance. Furthermore, since AMPs usually target bacterial cell walls and cell membranes that bacteria typically cannot modify without endangering their fitness, targeting crucial cell walls or cell membrane components likely also adds to the long-term efficiency of gut AMPs. 

## 6. Antimicrobial Stewardship and Modulation of Gut AMPs as a Tool to Fight against Resistance Development

The rationalized use of antibiotics is an important aspect of fighting against antimicrobial resistance by maintaining gut homeostasis and reducing alterations to gut AMPs. The rationalized use of antibiotics includes the choice, dose, and duration of antibiotic therapy. It has been reported in several large meta-analysis studies that using antimicrobial stewardship programs resulted in a reduced number of infections with MAR organisms [84,85]. The type and spectrum of antibiotics employed are critical factors in the development of resistance in the targeted microorganisms. The majority of the commensal population is anaerobic; thus, inappropriate and extended usage of anti-anaerobic antibiotics has been linked to an increased risk of MAR [86,87]. It has been reported that the use of narrow-spectrum antibiotics in place of anti-anaerobic antibiotics is favorable to the human gut microbiota since fewer commensals are impacted [88,89]. Further, the duration of antibiotic therapy has a direct impact on gut microbiota composition as it has been reported that shorter antibiotic courses result in fewer microbial disturbances and quicker gut microbiota restoration [88,90]. Moreover, the right dose of antibiotic is very important as lower doses are linked with less chance of resistance gene development; however, lower doses for extended periods can also cause resistance [91,92]. 

Interestingly, gut AMPs have great promise as innovative therapeutic antibiotics because they do not easily develop bacterial resistance. The broad development of AMPs as medicines, however, has been hampered by several factors. First, AMPs can have relatively short half-lives because they are extremely sensitive to proteolytic breakdown by microbial and host enzymes. Second, many AMPs are harmful to the membranes of eukaryotic cells and display cytotoxicity. 

Protein engineering techniques can be used to improve the bioavailability or efficacy of the AMPs because of their proteinaceous nature. It is possible to generate AMP versions that are resistant to enzymatic digestion. Also, using engineering peptidomimetics, new variants of AMPs could be generated with an altered number of charged amino acid residues with decreased hydrophobicity and cytotoxicity as well [93]. Additionally, the majority of AMPs kill bacteria by direct interaction with bacterial membranes. Interestingly, D-entantiomers of AMPs have longer half-lives and are just as effective at penetrating membranes as their natural L-entantiomers, so they can be used to improve the therapeutic efficacy of AMPs [94]. Further, packaging and delivering natural AMPs or their peptidomimetic analogs via nanoparticles can minimize non-specific cytotoxicity and improve stability with targeted bioavailability [95]. Additionally, novel AMPs should be employed for in vivo screening because the actual gut environment is completely different with the presence of different interactions with other commensals and their secreted AMPs which are already present in the gut. It is worth investigating the efficacy of new AMPs in the real dynamic gut environment against pathogenic bacteria or in conjunction with conventional antibiotics. Further new animal models with a controlled gut environment can be employed to check AMP efficacy in combination with antibiotics. Additionally, the gut has a diverse microbial ecology that differs for each individual. It is important to fully understand the gut microbial ecology for a detailed understanding of the interaction of gut AMPs with conventional antibiotics in the presence of other eukaryotic organisms including viruses, bacteriophages, and fungi. The interaction of gut AMPs with these diverse ecological community members individually or as a whole should be considered to understand their impact on gut AMP evolution and resistance development. Next-generation sequencing, transcriptomics, and gene expression analysis can further elucidate the mechanistic overview of complex gut environments that sheds light on unanswered questions and will further help in the development of a strategy to fight against resistance development. 

## 7. Conclusions and Future Perspective

Combinatory use of AMPs produced by both host and gut microbiota with conventional antibiotics could result in synergistic actions in different ways. It has been predicted that every species contains a unique set of AMPs that are evolved to defend the host against the microorganisms they might encounter [96]. This phenomenon becomes more complex and functionally specific in the case of the gut. The human gut is inhabited by millions of commensals which constitute the specific set of bacteria for every individual that is further affected by dietary habits, environment, and many more factors. Interestingly, there is a highly competitive environment in the gut so gut microbes are known to produce AMPs with various biological activities including immunomodulatory activities. Along with AMPs produced by gut microbiota, there are multiple host AMPs secreted in the gut in the proximity of gut epithelium and gut microbiota. It is hypothesized that all the gut AMPs synergistically affect each other’s functions to drive complex gut functions such as regulation of gut homeostasis; however, the mechanisms of this are not fully understood. In addition to fighting against infectious pathogens, gut AMPs play an essential role in the regulation of bacterial symbionts and communities in the gut, thus maintaining a balance between health and pathogenic microbes [97]. Further, gut microbiota exhibit high intrinsic resistance to AMPs which suggests that gut AMPs could be a customizable tool to maintain healthy gut communities [98]. Additionally, recent accumulating pieces of evidence suggest a functional synergism among the different gut AMPs [99]. The gut synergism may also reduce the chances of resistance evolution. Further, the synergistic mechanisms of gut AMPs could be used effectively in combination with conventional antibiotics to combat MAR. Another factor is that the host regulates the gut AMPs synergistically in such a way that limits the chances of rapid resistance evolution. These synergistic strategies could be further used for the effective translation of AMPs alone or in combination with conventional antibiotics into therapeutic applications. 

The human gut and AMP-producing intestinal epithelium constantly face a challenging dynamic microbial environment and also produce various antimicrobials for their survival that eventually affect the overall gut immune response including the efficacy of antibiotics during infection. To meet this challenge of the dynamic microbiome of the gut, epithelial cells also produce a wide variety of AMPs that quickly kill or inactivate bacteria, while a similar action is performed by the gut commensals to maintain the healthy gut environment which is called homeostasis. However, how the gut immune system differentiates between the healthy and pathogenic microbiota is still not well understood and remains a question of further research. On the other hand, in addition to this internal healthy equilibrium within the gut immune system, antibiotic treatment during infection creates another challenge for gut homeostasis. While both gut epithelium and commensals bear the adverse effects of antibiotics, gut AMPs have enough of a chance to interact with antibiotics, which affects the treatment efficacy as well (Table 2). However, it is not clear how AMPs interact with antibiotics and what is the response of AMP-producing gut epithelium and commensals in this dynamic complex gut environment. The emerging picture is that epithelial AMPs influence the structure and location of gut commensals in addition to protecting against pathogen colonization and invasion in synergism with the AMPs produced by commensals. Overall, gut AMPs are evolved for their antimicrobial action, efficacy, and spectrum under synergistic co-evolution with host immunity and commensals, along with interactions with other AMPs and conventional antibiotics (Figure 2). 

Finally, MAR resistance is rapidly growing while the discovery and availability of new antimicrobials are slow which generates an urgent demand for new antimicrobials along with a fully elucidated mechanism of resistance to overcome this crisis. At this point, the dissection of the gut microbiome as an antimicrobial resistance reservoir is much needed. This can be achieved by clinical and translational studies exploring the interaction of gut microbiome and gut AMPs within the gut microbial ecology and with conventional antibiotics. Functional metagenomic studies might be very helpful in identifying the uncultivable gut microbes and their role in resistance development and evolution. 

## 8. Unanswered Questions about Gut Microbiota and Gut AMPs

- What makes the gut microbiome healthy and what are the deciding bio-markers?
- What is the genetic machinery that regulates the production of gut AMPs?
- How do gut AMPs play a role in resistance development?
- Could diet help in the fight against resistance by manipulating gut microbiota? How?
- How do gut AMPs regulate the immune response to fight against resistance?
- How to reconstruct the gut microbiome and gut AMPome to counter antibiotic resistance?

## Figures and Tables

**Table 1:** Gut microbiota-produced AMPs and involvement in the treatment of different diseases.

Gut AMPs	Producing Bacteria	Targeted Pathogens or Diseases	References
Bacteriocin Abp118	*L. salivarius*	Listeriosis	[30]
Bacteriocin OR-7	*L. salivarius* NRRLB	*Campylobacter jejuni*	[31]
Bactofencin A	*L. salivarius*	Antilisterial, antistaphylococcal	[32]
Lactocin AL705	*L. curvatus*	Listeriosis	[33]
Lactocin 160	*L. rhamnosus*	*Escherichia coli* *Bordetella pertussis*	[34]
Lacticin3147	*Lactococcus lactis* DPC3147	*C. difficile*-associated diarrhea (CDAD)	[35]
Garvicin ML	*L. garvieae*	*Streptococcus pneumonia*	[36]
Nisin Z	*L. lactis*	Immunomodulatory effect	[37]
Nisin F	*L. lactis*	Respiratory infection	[38]
Nisin	*L. lactis*	Meningitis, sepsis, pneumonia	[39]
Nisin Z	*L. lactis*	Enteric pathogens	[37]
Nisin A	*L. lactis*	Colorectal cancer	[40]
Pediocin PA1	*Pediococcus acidilactici*	Listeriosis	[41]
Pediocin AcH	*P. acidilactici*	Enteric pathogens	[37]
Enterocin CRL35	*Enterococcus mundtii* RL35	Listeriosis	[42]
Avicin	*E. avium*	Listeriosis	[43]
Enterocin P	*E. faecium* P13	Enteric pathogens	[44]
Piscicolin 126, carnobacteriocin	*Carnobacterium maltaromaticum*	Listeriosis	[45]
Kimchichin	*Leuconostoc citreum* GJ7	*Salmonella typhi*	[46]
Erwinaocin NA4	*Erwinia carotovora* NA4	Coliphage	[47]

**Table 2:** Gut AMPs in synergy with conventional antibiotics.

Gut AMPs	Antibiotics	Target	References
Nisin	Ramoplanin	MRSA	[74]
	Polymyxin E Clarithromycin	*P. aeruginosa*	[75]
	Amoxicillin Penicillin Streptomycin Ceftiofur Tetracycline	*S. suis*	[65]
Nisin Z	Ampicillin Chloramphenicol Kanamycin Lincomycin Penicillin G Rifampicin Streptomycin Tetracycline Vancomycin	*P. fluorescens* LRC-R73 and its Penicillin-resistant/Streptomycin-resistant/Lincomycin-resistant/Rifampicin-resistant variant	[63]
Lacticin 3147	Polymyxin B	*S. aureus* 5247	[76]
Actagardine	Ramoplanin Metronidazole Vancomycin	*C. difficile*	[77]
Thuricin CD	Ramoplanin	*C. difficile*	[77]
	Vancomycin	*C. difficile*	[77]
Subtilosin A	Clindamycin phosphate Metronidazole	*G. vaginalis*	[66]
	Lauramide arginate Ester poly-lysine	*G. vaginalis*	[66]
PsVP-10	Chlorhexidine	*S. mutans* *S. sobrinus*	[78]
Plantaricin E, F, J, K	Several antibiotics	*C. albicans*	[79]
Colistin	Tobramycin	*P. aeruginosa*	[73]
Cryptdin 2	Ampicillin	*S. typhimurium*	[68]
Laterosporulin10	Rifampicin	*M. tuberculosis* H37Rv	[62]
Colistin	Azithromycin	*A. baumannii* *K. pneumoniae* *P. aeruginosa*	[71]
LL-37	Azithromycin	*A. baumannii* *K. pneumoniae* *P. aeruginosa*	[71]
Human defensin 5 (HD5)	Meropenem	*C. difficile*	[67]
Human neutrophil peptide-1 (HNP1)	Rifampicin	*M. tuberculosis* H37Rv	[69]
Human β-defensin 3 (HBD3)	Meropenem Moxifloxacin Piperacillin-Tazobactam Tigecycline	*C. difficile*	[67]
